# Unravelling targeted therapy in prostate cancer: from molecular mechanisms to translational opportunities

**DOI:** 10.3389/fcell.2025.1685857

**Published:** 2025-11-14

**Authors:** Litong Wu, Junfeng Qiu, Zhiming Hong, Quan Wang, Qixin Li, Wenbin Zhou

**Affiliations:** 1 Andrology Department, Shenzhen Traditional Chinese Medicine Hospital, Shenzhen, China; 2 The Fourth Clinical Medical College, Guangzhou University of Chinese Medicine, Shenzhen, China; 3 Shenzhen Bao’an Traditional Chinese Medicine Hospital Group, Shenzhen, China

**Keywords:** prostate cancer, targeted therapy, PROTACs, androgen receptor, tumor microenvironment, molecular mechanisms

## Abstract

Prostate cancer, ranking among the most prevalent malignancies in males worldwide, is undergoing a significant evolution in therapeutic paradigms from conventional approaches to precision medicine, with recent advances in targeted therapies offering novel strategic insights. This review delineates the molecular foundations of prostate carcinogenesis, elucidating pivotal domains including genetic mutations, hormonal regulation, tumor microenvironment dynamics, cell cycle dysregulation, epigenetic modifications, and tumor heterogeneity. Furthermore, we evaluate the clinical translation of targeted strategies such as AR signaling axis inhibition, PI3K/AKT/mTOR pathway modulation, DNA damage repair machinery exploitation, prostate-specific membrane antigen -directed interventions, and combinatorial immunotherapy. Concurrent challenges—AR-driven heterogeneity, adaptive drug resistance mechanisms, spliceosomal vulnerabilities, and scarcity of selective molecular targets—are critically analyzed. Notwithstanding these obstacles, targeted therapies exhibit considerable potential to enhance therapeutic efficacy while mitigating systemic toxicities, paving the way for more personalized and precision-oriented oncologic care. By underscoring the imperative to decode prostate cancer’s molecular architecture, this work outlines future research priorities and advances a robust scientific framework for innovation in therapeutic development.

## Graphical Abstract

**Figure:**
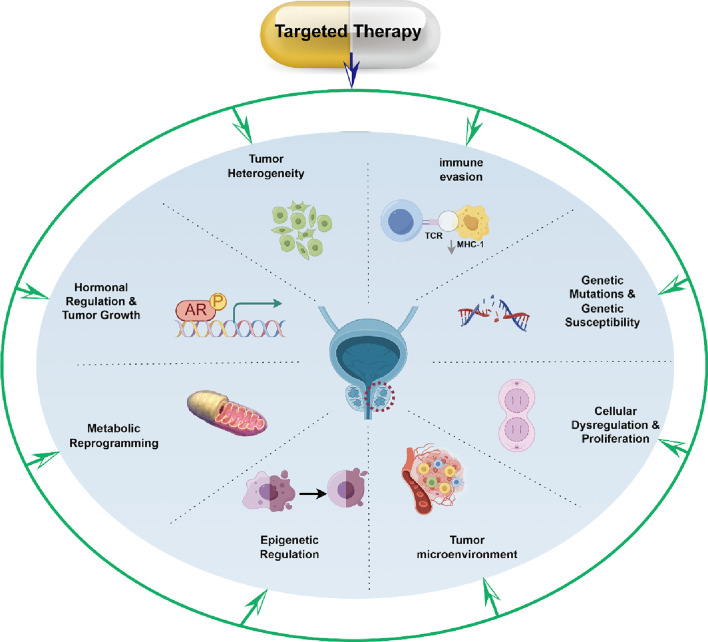

## Highlights

- PROTACs degrade resistant AR variants–Novel AR degraders (e.g., ARV-110) overcome castration resistance in clinical trials.
- PSMA theranostics redefine mCRPC management–^225^Ac-J591 achieves 46.9% PSA50 response with targeted alpha therapy.
- PARP-ICI synergy exploits DDR defects–Olaparib/durvalumab combinations induce immunogenic death in HRR-deficient tumors.
- Molecular stratification guides precision therapy–BRCA2 (56.6%), MSI-H, and AR-V7 serve as actionable biomarkers.
- TME reprogramming reverses immunosuppression–AR inhibition synergizes with ICIs by downregulating PD-L1/Tregs.

## 1 Introduction

Prostate cancer (PCa), one of the most prevalent solid malignancies among men worldwide, represents a leading cause of male cancer-related mortality (Sung et al., 2021; Bergengren et al., 2023). For localized early-stage PCa, therapeutic options include radical prostatectomy, external beam radiotherapy, and androgen deprivation therapy (ADT), while advanced or metastatic disease typically necessitates multimodal approaches combining ADT with chemotherapy and radiation (Li et al., 2024). ADT has remained the cornerstone of PCa management for over seven decades, demonstrating unparalleled efficacy in disease control (Nabavi et al., 2023; Wang et al., 2023). However, both surgical and pharmacological castration inevitably culminate in therapeutic resistance (Vigneswaran et al., 2021). Castration-resistant prostate cancer (CRPC) emerges as the terminal trajectory for most patients, characterized by dismal clinical outcomes, with metastatic CRPC (mCRPC) exhibiting a median overall survival (OS) of less than 2 years (Lowrance et al., 2018). A significant proportion of patients with CRPC develop resistance to prior ADT or chemotherapy and experience systemic toxicities, accompanied by rising prostate-specific antigen (PSA) levels, AR mutations, and aberrant RNA transcription. Consequently, their survival benefit is typically less than six months—a stark imperative for novel therapeutic interventions (Jones et al., 2020; Kour et al., 2023; Carranza-Aranda et al., 2024; Lv et al., 2024).

Targeted therapy, an innovative oncologic strategy, operates through precise identification and engagement of tumor-specific molecular targets, diverging from conventional therapies that indiscriminately affect rapidly dividing cells. This approach offers superior selectivity, minimized off-target toxicity, and enhanced therapeutic precision (Qian et al., 2020; Pham et al., 2021; Viktorsson et al., 2023). The advent of targeted therapies has contributed to a shift in oncology—from traditional histology-driven chemoradiotherapy paradigms to molecularly informed personalized approaches. Building on this framework, this review synthesizes recent advancements in PCa-targeted therapeutics, encompassing molecular pathogenesis, contemporary pharmacologic agents, and innovative strategies, while providing a critical appraisal of persistent challenges and emerging countermeasures in this rapidly evolving field.

## 2 Molecular pathogenesis of prostate cancer

**FIGURE 1:**
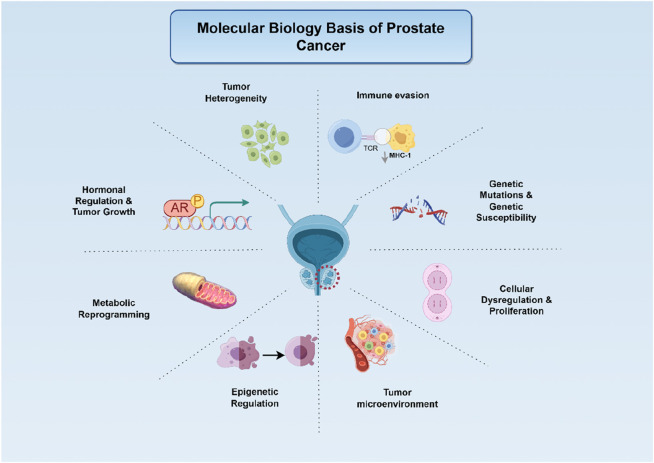
Biological mechanisms underlying prostate carcinogenesis and progression (Legend: Red circular dashed line: The location of PCa. Arrows: Activating or promoting effects. From top to bottom: (1) Tumour heterogeneity arises through clonal selection, generating sub-populations with distinct genomic/epigenomic profiles. (2) Immune evasion mechanisms allow tumour cells to escape immune surveillance. (3) Hormonal regulation centred on AR signalling supports tumour cell survival and proliferation. (4) Inherited genetic susceptibility and sporadic mutations destabilise the genome. (5) Metabolic reprogramming (aerobic glycolysis, lipid synthesis) fuels biomass production and redox balance. (6) Cellular Dysregulation and Proliferation is driven by cell-cycle checkpoint loss that trigger unchecked prostate-cancer cell division. (7) Epigenetic alterations (DNA methylation, histone modifications) silence tumour-suppressor genes and activate oncogenes. (8) The altered tumour microenvironment further promotes growth and metastasis).

PCa represents a multifactorial disorder driven by intricate genetic and molecular alterations, as illustrated in Figure 1. A comprehensive understanding of its molecular underpinnings is pivotal for advancing targeted therapeutic strategies.

### 2.1 Genetic mutations and hereditary predisposition

Genetic mutations and hereditary susceptibility serve as critical determinants in PCa pathogenesis. Table 1 summarizes the frequency of gene mutations closely associated with PCa. Among these, BRCA1/2 mutations—originally linked to breast and ovarian cancers—have emerged as significant risk amplifiers for PCa (Abida et al., 2020; Boussios et al., 2022; Fettke et al., 2023). These genes encode proteins essential for homologous recombination repair (HRR) of DNA double-strand breaks; their dysfunction leads to genomic instability and carcinogenesis. Chen et al. characterized BRCA germline mutations in Chinese PCa cohorts, analyzing 172 patients with BRCA1/2 alterations (Chen et al., 2022). The cohort exhibited a median diagnosis age of 67 (range: 34—89), with 54.65% (94/172) presenting metastatic castration-resistant disease, indicative of aggressive biology. Frameshift, missense, and splice variants predominated, with BRCA2 mutations surpassing BRCA1 in frequency. Notably, HOXB13, MSH2, and MSH6 mutations further contribute to PCa susceptibility.

**TABLE 1:** The proportion of important gene mutations related to prostate cancer.

BRCA1+	BRCA2+	AR+	TP53+	FOXA1+
17.46% (11–63)	56.55% (82/145)	15% (9/59)	15% (9/59)	34% (20/59)

HOXB13, a homeobox transcription factor critical in embryogenesis and tissue homeostasis, harbors pathogenic variants strongly associated with hereditary PCa (Nyberg et al., 2019). Mechanistically, Lu et al. demonstrated that HOXB13 recruits HDAC3 to suppress *de novo* lipogenesis and metastasis, while its loss or mutation drives lipid accumulation, enhancing tumor cell motility and metastatic potential (Lu et al., 2022). These findings suggest therapeutic utility of lipogenic pathway inhibitors in HOXB13-deficient PCa. MSH2 and MSH6, core components of DNA mismatch repair (MMR), safeguard replication fidelity. Their inactivation induces microsatellite instability (MSI), a biomarker of immunotherapy responsiveness. Wyvekens et al. evaluated 19 MMR-deficient PCa cases, identifying MSH2/MSH6 loss as the predominant defect, with distinct histopathological features aiding diagnostic recognition (Wyvekens et al., 2022).

### 2.2 Hormonal regulation and neoplastic progression

**FIGURE 2:**
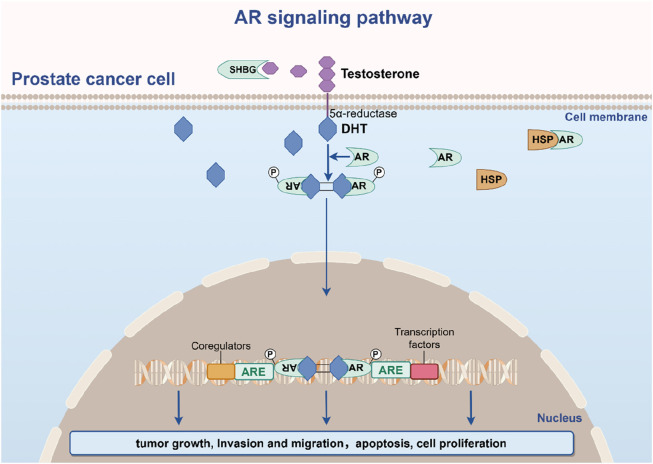
The AR signaling pathway in prostate cancer pathogenesis.

Androgen signaling, mediated via the androgen receptor (AR), remains central to PCa biology (Figure 2). Testosterone and its potent metabolite dihydrotestosterone (DHT) bind AR, a steroid receptor comprising four domains: N-terminal transcriptional regulation, DNA-binding, hinge, and ligand-binding. In unliganded states, AR resides in the cytoplasm, chaperoned by HSP90/70 complexes (Likos et al., 2022; Knerr et al., 2023). Ligand binding triggers conformational changes, nuclear translocation, dimerization, and DNA binding to androgen response elements, driving transcription of genes that promote proliferation, survival, and metastasis (Xie et al., 2022; Özturan et al., 2022; Sun et al., 2023).

Early-stage PCa exhibits androgen dependence, making AR pathway inhibition a cornerstone of therapy for locally advanced or metastatic disease. However, adaptive mechanisms—AR amplification, gain-of-function mutations, splice variant generation (e.g., AR-V7), and downstream signaling rewiring—culminate in CRPC (Formaggio et al., 2021; Isebia et al., 2023). Beyond intrinsic tumor cell effects, androgens modulate the tumor microenvironment (TME) by polarizing tumor-associated macrophages, activating cancer-associated fibroblasts, suppressing immune surveillance, and stimulating angiogenesis (Hahn et al., 2023). Deciphering these multidimensional interactions is critical for identifying novel therapeutic vulnerabilities in PCa’s evolving landscape.

### 2.3 Tumor microenvironment and immune evasion

The TME and immune evasion mechanisms play pivotal roles in PCa progression. The TME constitutes a dynamic ecosystem comprising cancer cells, immune cells (e.g., tumor-associated macrophages (TAMs), regulatory T cells (Tregs), natural killer (NK) cells), stromal fibroblasts, vascular networks, and extracellular matrix components. This milieu not only sustains tumor survival but also orchestrates immune evasion through multifaceted interactions (Kwon et al., 2021; Wong et al., 2022; Hirz et al., 2023). TAMs, particularly lipid-laden subsets, drive PCa invasiveness via IL-1β-mediated upregulation of MARCO, which reciprocally triggers CCL6 secretion to enhance cancer cell migration (Masetti et al., 2022). Tregs amplify immunosuppression by releasing TGF-β and IL-10, establishing an immune-tolerant niche linked to elevated recurrence risk (Karpisheh et al., 2021). Paradoxically, NK cells and tumor-infiltrating lymphocytes (TILs) exhibit dual roles—suppressing tumor growth or being co-opted to facilitate immune escape (Pasero et al., 2016; Ocana et al., 2017).

PCa cells employ multifaceted immune-editing mechanisms to evade immune surveillance, fostering clonal selection of immunoresistant subpopulations. Key immunosuppressive strategies involve the secretion of specific ligands and cytokines—such as PD-L1, TGF-β, and IL-10—which inhibit T-cell activation and promote Tregs expansion (Zhu et al., 2023). Additionally, PCa cells recruit inhibitory immune cells including myeloid-derived suppressor cells and M2-polarized TAMs via chemokine signaling (Wu et al., 2022). These cells further amplify immunosuppression through arginase-1, iNOS, and reactive oxygen species production, effectively dampening cytotoxic T-cell responses. Concurrently, downregulation of major histocompatibility complex class I molecules impairs antigen presentation, enabling tumor cells to evade CD8^+^ T-cell recognition. These processes collectively establish an immunosuppressive TME that shields tumors from cytotoxic immune responses, posing formidable therapeutic challenges.

### 2.4 Cell cycle dysregulation and proliferative signaling

Dysregulated cell cycle control is a hallmark of PCa pathogenesis. Normally governed by stringent checkpoints to ensure genomic fidelity, the cell cycle becomes hijacked in PCa through aberrant activation of proliferative pathways and inactivation of tumor suppressors. PTEN, a critical phosphatase, constrains PI3K/AKT/mTOR signaling to inhibit uncontrolled growth. Its frequent loss in PCa leads to constitutive AKT activation, NF-κB-driven stemness, and evasion of growth suppression (Dubrovska et al., 2009; Kim et al., 2014). Concurrently, p53 dysfunction—via mutation or epigenetic silencing—compromises DNA damage response, enabling survival of genomically unstable clones (Macedo-Silva et al., 2023).

MYC proto-oncogene overexpression further disrupts cell cycle governance by antagonizing AR-mediated transcriptional programs and bypassing AR-dependent transcriptional pausing. This drives S-phase entry through upregulation of ribosome biogenesis genes and cyclin-dependent kinases, accelerating proliferation while fostering genomic instability (Qiu et al., 2022). The interplay between PTEN/PI3K/AKT, p53, and MYC pathways creates a complex regulatory nexus, complicating therapeutic targeting and underscoring the need for combinatorial strategies to address convergent oncogenic networks.

### 2.5 Epigenetic regulation and metabolic reprogramming

Epigenetic mechanisms—including DNA methylation, histone modifications, and non-coding RNA-mediated regulation—orchestrate PCa pathogenesis by modulating gene expression patterns without altering genomic sequences. These processes drive tumor progression, metastasis, and therapeutic resistance through transcriptional silencing or activation of critical pathways. Hypermethylation of tumor suppressor genes, exemplified by GSTP1 inactivation in PCa, disrupts detoxification mechanisms and potentiates carcinogen-induced DNA damage, as evidenced by a meta-analysis of 15 studies (Zhou et al., 2019; Zhao et al., 2020). Concurrently, histone acetylation/methylation dynamically remodels chromatin architecture to either enhance oncogenic transcription or repress tumor-suppressive programs (Metzger et al., 2019; Topchu et al., 2022; Nguyen et al., 2023).

Metabolic reprogramming represents an adaptive strategy for PCa cells to meet biosynthetic and energetic demands. Unlike normal prostate epithelium, PCa exhibits heightened lipogenesis and a pronounced Warburg effect—preferential glycolysis despite oxygen availability—to fuel rapid proliferation and therapy resistance (Lai et al., 2023). This metabolic shift is bidirectionally linked to epigenetic regulation: epigenetic modifiers directly control metabolic enzyme expression, while metabolites such as α-ketoglutarate and S-adenosylmethionine serve as cofactors for histone/DNA-modifying enzymes. Such crosstalk enables dynamic adaptation to microenvironmental stressors, fostering tumor survival and dissemination.

### 2.6 Tumor heterogeneity and evolutionary dynamics

PCa progression is defined by multidimensional heterogeneity—interpatient (intertumoral), intratumoral, and cellular—arising from clonal evolution under selective pressures. This diversity, driven by stochastic mutations, epigenetic plasticity, metabolic adaptations, and microenvironmental gradients, underpins therapeutic failure and relapse (Haffner et al., 2021; Chakraborty et al., 2023). Exome sequencing of 37 samples from 16 PCa patients revealed recurrent alterations in DNA damage repair (DDR) genes, RTK/RAS pathway components, and autophagy regulators, with copy number variation burden correlating with metastatic potential (Wu et al., 2020). Spatial heterogeneity in oxygen tension and nutrient availability further selects for clones optimized for survival in hypoxic or nutrient-deprived niches (Peitzsch et al., 2022).

The TME acts as both a driver and consequence of heterogeneity, fostering competitive interactions between clones with divergent genetic, epigenetic, and metabolic profiles. This evolutionary arms race necessitates polytherapeutic strategies targeting core vulnerabilities across heterogeneous subpopulations to mitigate adaptive resistance.

## 3 Current targeted therapeutics and clinical strategies

Targeted therapies have revolutionized the management of PCa, offering patients more precise and effective treatment options. By specifically targeting key molecules and pathways driving tumor growth and dissemination, these therapies minimize damage to normal cells, achieving superior therapeutic efficacy and reduced systemic toxicity compared to conventional approaches. In PCa, therapeutic focus centers on critical biomarkers such as the AR, proliferative signaling cascades, and DNA repair mechanisms. Advances in basic research and clinical trials continue to expand the pipeline of targeted agents and combination strategies, heralding a new era of innovation in PCa therapeutics. Current investigational agents under clinical evaluation are summarized in Table 2.

**TABLE 2:** List of drug information during clinical trials.

Trial identification	Drug name	Target	Trial phase	No. of patients	Target disease (prior therapy)
CTR20150501	GT0918	AR	phase I	16	CRPC (Chemotherapy failure)
NCT02861573	Pembrolizumab	PD-1	phase Ib/II	102	CRPC (ADT failure)
NCT02361086	ODM-204	CYP17A1/AR	phase I	23	CRPC (ADT failure)
NCT02709889	Rovalpituzumab tesirine (SC16LD6.5)	AR	phase II	99	CRPC (ADT failure)
NCT03888612	Bavdegalutamide	AR	phase I/II	195	mCRPC (ADT failure)
NCT02121639	Capivasertib	AKT	phase II	150	CRPC (Chemotherapy failure)
NCT04087174	Capivasertib	PI3K/AKT/mTOR	phase Ib	27	nmCRPC (ADT failure)
NCT02407054	Samotolisib	PI3K and mTOR	phase Ib/II	13/129	mCRPC (ADT failure)
NCT03017833	Sapanisertib (CB-228/TAK-228)	mTORC1/2	phase I	30	PCa (ADT failure)
NCT02215096	GSK2636771	PI3Kβ	phase I	37	CRPC (ADT failure)
NCT03317392	Olaparib	PARP	phase I	12	mCRPC (ADT failure)
NCT04169841	Olaparib	PARP	phase II	213	PCa (ADT failure)
NCT03431350	Niraparib	PARP	phase II	24	mCRPC (ADT failure)
NCT02924766	Niraparib	PARP1/2	phase Ib	33	mCRPC (ADT failure)
NCT02854436	Niraparib	PARP1/2	phase II	289	mCRPC (ADT failure)
NCT03276572	^225^Ac-J591	PSMA	phase II	32	mCRPC (Chemotherapy or ADT failure)
NCT03999749	JNJ-63898081	PSMA	phase I	39	mCRPC (Chemotherapy or ADT failure)
NCT02484404	Olaparib + durvalumab	PARP + PD-L1	phase II	17	mCRPC (Chemotherapy or ADT failure)
NCT03016312	Enzalutamide + Atezolizumab	AR + PD-L1	phase Ⅲ	759	mCRPC (ADT failure)
NCT03805594	177Lu-PSMA-617+pembrolizumab	PSMA + PD-1	phase I	43	mCRPC (ADT failure)

### 3.1 Targeting the androgen receptor signaling pathway

#### 3.1.1 Clinical applications of second-generation antiandrogens and emerging agents

The AR signaling axis plays a central role in PCa initiation and progression. While ADT remains a mainstay by suppressing AR activity, long-term treatment inevitably leads to resistance (Obinata et al., 2024). Recent discoveries of novel AR-associated targets have spurred the development of next-generation antiandrogens. Second-generation agents such as enzalutamide and abiraterone inhibit AR signaling through distinct mechanisms, demonstrating robust antitumor activity and improved clinical outcomes in mCRPC (Mitsogianni et al., 2023; Obinata et al., 2024). Nevertheless, resistance persists in a subset of patients, driving exploration of novel AR-targeted strategies.

A phase I trial evaluated GT0918, a novel AR antagonist, in 16 patients with mCRPC across five escalating dose cohorts (Zhou et al., 2020). Ten and two patients completed three and six treatment cycles, respectively. Six patients achieved ≥30% PSA decline, with two attaining ≥50% reduction. Stable disease was observed in all 12 patients with metastatic soft tissue lesions. GT0918 demonstrated high AR binding affinity, downregulation of AR protein expression, and favorable tolerability, suggesting promising antitumor activity in the CRPC population.

Combination strategies leveraging multi-target inhibition are gaining momentum. ODM-204, a dual CYP17A1/AR inhibitor, was tested in a clinical trial where 13% of patients achieved ≥50% PSA reduction by week 12, with 60.9% experiencing mild treatment-related adverse events (Peltola et al., 2020). ODM-204 was well-tolerated, with preliminary antitumor activity observed in mCRPC. In a preclinical study, Baker et al. developed a combinatorial nanotherapeutic platform—abiraterone-enzalutamide bio-conjugated survivin-encapsulated gold nanoparticles (AbEzSvGNPs)—for targeted PCa therapy (Baker et al., 2023). Compared to free abiraterone and enzalutamide, AbEzSvGNPs exhibited enhanced cytotoxicity against DU145(IC_50_ = 4.21 μM) and PC-3(IC_50_ = 5.58 μM) cells while showing no significant toxicity in normal rat kidney cells.

#### 3.1.2 Advances in PROTAC-Based targeted therapies

Proteolysis-targeting chimeras (PROTACs) represent a novel therapeutic modality in PCa, leveraging the ubiquitin-proteasome system to selectively degrade pathogenic proteins—a mechanism distinct from traditional small-molecule inhibition (Wang et al., 2025). PROTACs are heterobifunctional molecules comprising three components: a target protein ligand, an E3 ubiquitin ligase recruiter, and a linker. By bridging the target protein with an E3 ligase, PROTACs induce ubiquitination and subsequent proteasomal degradation of the target (Zeng et al., 2021). This approach has garnered significant attention in oncology, particularly for addressing resistant AR variants and castration-resistant AR signaling in PCa.

ARV-110 (bavdegalutamide), the first PROTAC to enter clinical trials, is currently in phase II evaluation for mCRPC. ARV-110, an orally bioavailable, CRBN-based AR degrader developed by Arvinas, Inc., demonstrated promising efficacy in a phase I/II trial. It reduced PSA levels by ≥ 50% in 40% of patients with mCRPC harboring specific genetic alterations. Furthermore, in initial clinical studies, biopsy data from one patient showed a 70%–90% reduction in AR levels (Liu et al., 2022). Malarvannan et al. highlighted the potential of PROTACs to overcome drug resistance and target “undruggable” proteins, citing ARV-110 and ARV-766 (another AR-directed PROTAC in phase II trials for CRPC) as exemplars (Malarvannan et al., 2025). Omar et al. reviewed advancements in PROTAC design, proposing the use of heterocyclic compounds as warheads to optimize binding affinity, selectivity, and pharmacokinetic properties (Omar et al., 2025). This structural refinement enhances PROTAC efficacy, positioning them as promising tools for addressing persistent challenges in PCa therapy.

### 3.2 Targeting the PI3K/AKT/mTOR signaling axis

The PI3K/AKT/mTOR pathway, a critical oncogenic cascade, drives PCa progression by promoting tumor cell proliferation, migration, and therapeutic resistance through aberrant activation (Pungsrinont et al., 2021; Wylaź et al., 2023; Yi et al., 2023). Multiple PI3K, AKT, and mTOR inhibitors have entered clinical trials, demonstrating variable antitumor efficacy. Emerging next-generation inhibitors aim to enhance therapeutic precision while minimizing adverse effects.

Capivasertib, a pan-AKT inhibitor, exhibits synergistic activity with docetaxel in mCRPC. In a randomized phase II trial involving 150 mCRPC patients receiving up to 10 cycles of docetaxel (21-day cycles), capivasertib combined with chemotherapy prolonged OS, though these findings require prospective validation to address potential biases (Crabb et al., 2021). A phase Ib study further evaluated capivasertib (400 mg twice daily, 4 days on/3 days off) combined with abiraterone acetate (1,000 mg daily) and prednisone (5 mg twice daily) in mCRPC. Nine patients (33%) achieved ≥20% PSA decline, with no dose-limiting toxicities observed, supporting further investigation of this regimen (Shore et al., 2023).

Samotolisib, a dual PI3K/mTOR inhibitor employing intermittent target suppression, demonstrated enhanced tolerability and delayed resistance in a blinded, placebo-controlled Ib/II trial (Sweeney et al., 2022). Phase Ib (n = 13) revealed no dose-limiting toxicities, while phase II (n = 129) showed significantly prolonged median progression-free survival (PFS) and radiographic PFS(rPFS) in the samotolisib/enzalutamide arm versus placebo. This underscores the feasibility of combining PI3K/mTOR inhibition with AR-targeted therapy.

Subbiah et al. explored sapanisertib, an ATP-competitive mTORC1/2 inhibitor, combined with metformin in patients with mTOR/AKT/PI3K pathway-altered advanced malignancies (Subbiah et al., 2024). The combination exhibited tolerable safety and antitumor activity, particularly in PTEN-mutated cohorts. Metformin’s AMPK-mediated mTOR suppression may potentiate sapanisertib’s efficacy, offering a rationale for dual metabolic-oncogenic targeting in PCa. A phase I dose-escalation study of GSK2636771(PI3Kβ inhibitor) with enzalutamide in PTEN-deficient mCRPC(n = 37) reported a 50% non-progression rate at 12 weeks with the recommended 200 mg dose, though objective responses remained limited (1 patient with 36-week partial response) (Sarker et al., 2021). These data highlight modest activity despite acceptable safety, emphasizing the need for biomarker-driven patient selection. In addition, bioactive phytochemicals, including flavonoids, terpenoids, alkaloids, lignans, phenolic acids, and polysaccharides, exhibit preclinical efficacy in PCa through selective modulation of the PI3K/AKT/mTOR pathway. These natural agents regulate downstream effectors to suppress tumor proliferation, induce apoptosis, and reverse therapeutic resistance, positioning them as promising candidates for adjunctive therapeutic modalities or complementary strategies in PCa management (Lu et al., 2020; León-González et al., 2021; Jeong et al., 2023; Elsayed and Fahim, 2025; Filippi et al., 2025).

### 3.3 Targeting DNA damage repair pathways

Dysregulation of DDR mechanisms is a hallmark of prostate carcinogenesis. Therapeutic strategies targeting these pathways have demonstrated clinical promise, particularly in genetically defined subsets of PCa. PARP inhibitors, such as olaparib and rucaparib, exploit synthetic lethality by impairing base excision repair in tumors with homologous recombination deficiency, notably those harboring BRCA1/2 mutations (Teyssonneau et al., 2021; Stracker et al., 2023).

A phase I dose-escalation study evaluated olaparib combined with radium-223 in mCRPC patients with bone metastases, establishing a recommended phase II dose (RP2D) of 200 mg twice daily for olaparib when administered with radium-223 (Pan et al., 2023). Niraparib (NIRA), a selective PARP1/2 inhibitor, was investigated in a phase II trial combining it with abiraterone acetate and prednisone in mCRPC patients progressing on androgen receptor signaling inhibitors (ARSIs) and taxanes (Chi et al., 2023). The regimen showed measurable antitumor activity and manageable toxicity, supporting further exploration. A phase Ib trial further assessed NIRA paired with apalutamide or abiraterone acetate/prednisone (AAP) in mCRPC, confirming tolerability and identifying NIRA 200 mg as the RP2D for combination with AAP (Saad et al., 2021). In a multicenter phase II study (n = 289), niraparib exhibited clinical activity in heavily pretreated mCRPC patients with DDR defects, particularly BRCA-mutated cohorts, reinforcing its therapeutic potential in biomarker-selected populations (Smith et al., 2022).

### 3.4 PSMA-targeted therapeutic innovations

Prostate-specific membrane antigen (PSMA), a transmembrane glycoprotein overexpressed in PCa with expression levels correlating to tumor aggressiveness, has emerged as a cornerstone for precision theranostics. Current PSMA-directed strategies encompass radioligand therapies (e.g., 177Lu-PSMA-617, 225Ac-PSMA-RLT), antibody-drug conjugates (MLN2704, PSMA-MMAE), cellular immunotherapies (PSMA-CAR-T, BiTEs), and experimental modalities such as photodynamic therapy and ultrasound-mediated nanobubble ablation. Radioligand therapies, characterized by high tumor specificity and reduced off-target toxicity, are increasingly prioritized for their ability to overcome tumor heterogeneity (Parghane and Basu, 2023; Desai et al., 2024; Ling et al., 2024; Nakajima, 2024; Ye et al., 2024; Belabaci et al., 2025). A phase I dose-escalation trial of 225Ac-J591, an α-emitting anti-PSMA monoclonal antibody, demonstrated preliminary efficacy in 32 patients with progressive mCRPC, with 46.9% achieving ≥50% PSA decline (34.4% confirmed) and 59.1% exhibiting circulating tumor cell control, alongside a manageable safety profile (Tagawa et al., 2024). At the final follow-up, disease progression and/or death had occurred in nearly all patients (29 out of 32). The median PFS was 5.6 months (95% CI, 3.7–7.9), and the median OS was 10.7 months. Similarly, a phase I study of JNJ-63898081 (JNJ-081), a PSMA-targeted agent, explored intravenous (0.3–3.0 µg/kg) and subcutaneous (3.0–60 µg/kg) administration in 39 mCRPC patients. While dose-limiting toxicities occurred in four cases, transient PSA reductions were observed at subcutaneous doses ≥30 µg/kg, suggesting therapeutic potential despite challenges such as cytokine release syndrome at higher doses (Lim et al., 2023). The integration of PSMA-PET/CT into clinical workflows has revolutionized diagnostic staging and restaging, enabling precise patient stratification for PSMA-directed therapies. However, the synergistic potential of combining PSMA-targeted approaches with standard treatments remains underexplored, necessitating further investigation to optimize combinatorial efficacy and safety. Advances in radiopharmaceutical engineering and imaging technologies are poised to refine therapeutic precision, offering renewed hope for metastatic PCa management through tumor-selective targeting and minimized systemic toxicity.

### 3.5 Combinatorial targeted and immunotherapeutic strategies in prostate cancer

The integration of targeted therapies with immunomodulatory agents represents an important strategy in PCa management. Targeted therapies disrupt oncogenic signaling by selectively inhibiting molecular drivers of tumorigenesis, while immunotherapies harness the host immune system to eradicate residual disease. This synergy is amplified by the ability of targeted agents to remodel the TME, enhance tumor antigen presentation, and potentiate immune effector cell activity, thereby overcoming limitations of monotherapy and improving therapeutic efficacy and tolerability (Zhu et al., 2021).

#### 3.5.1 PARP inhibitors and immune checkpoint blockade

The combination of PARP inhibitors with immune checkpoint inhibitors (ICIs) exploits dual mechanisms of synthetic lethality and immune activation. PARP inhibitors impair DNA repair via PARP enzyme blockade, inducing lethal DNA damage in homologous recombination repair (HRR)-deficient tumors (e.g., BRCA1/2-mutated PCa) (Wu et al., 2021). Concurrently, ICIs such as anti-PD-1/PD-L1 or anti-CTLA-4 agents reinvigorate T-cell-mediated antitumor responses, which are often suppressed in PCa(Catalano et al., 2022).

Crucially, the efficacy of this combinatorial strategy is highly dependent on the specific underlying DDR defect. A growing body of clinical evidence indicates that tumors harboring “BRCA1/2” mutations derive the greatest benefit. For instance, in the phase I/II Study study (n = 17), the combination of olaparib and durvalumab in mCRPC demonstrated a higher objective response rate (ORR) in patients with “BRCA1/2” alterations compared to those with other HRR gene mutations (Karzai et al., 2018).

A phase II trial evaluating durvalumab (anti-PD-L1) and tremelimumab (anti-CTLA-4) with olaparib in HRR-deficient solid tumors demonstrated synergistic immunogenic cell death and disease stabilization, supporting further exploration in PCa cohorts (Fumet et al., 2020). Meta-analyses of clinical trials reveal superior ORR, prolonged median progression-free survival, and significant PSA reductions with PARP-ICI combinations compared to monotherapy, alongside acceptable toxicity profiles (Mateo et al., 2015; Karzai et al., 2018; Antonarakis et al., 2020). However, increased risks of hematologic abnormalities, gastrointestinal toxicity, and immune-related adverse events necessitate vigilant monitoring and refined, biomarker-guided patient selection, prioritizing those with “BRCA1/2” mutations for the most robust clinical benefit (Hunia et al., 2022).

#### 3.5.2 AR pathway inhibition and immunotherapy synergy

AR inhibitors modulate the immunosuppressive TME by downregulating PD-L1 expression, reducing Tregs infiltration, and enhancing CD8^+^ T-cell functionality (Cordes et al., 2018; Dib et al., 2019). Preclinical studies demonstrate that AR blockade mitigates T-cell exhaustion and augments interferon-γ signaling, sensitizing tumors to PD-1/PD-L1 inhibition (Guan et al., 2022). Clinical trials, however, yield mixed outcomes. The KEYNOTE-365 Cohort C trial (Ib/II phase) reported limited antitumor activity for enzalutamide combined with pembrolizumab in chemotherapy-naïve mCRPC patients post-abiraterone failure, though safety profiles aligned with individual agent characteristics (Yu et al., 2024). Conversely, a phase III trial (n = 759) showed improved PFS in mCRPC patients with high PD-L1(IC2/3) and CD8^+^ gene expression treated with enzalutamide plus atezolizumab, though OS benefits were not observed (Powles et al., 2022). These findings underscore the need for biomarker-driven stratification and optimized dosing to address heterogeneous responses and mitigate immune-related toxicities.

#### 3.5.3 PSMA-targeted and immunotherapeutic convergence

PSMA-directed therapies synergize with immunotherapies through multimodal mechanisms: 1. Radioligand-induced immunogenic cell death: 177Lu-PSMA-617 and 225Ac-PSMA-RLT trigger tumor apoptosis and neoantigen release, enhancing immune recognition and dendritic cell activation (Pouget et al., 2023). 2. TME reprogramming: Radiation-induced DNA damage stimulates STING pathway activation and pro-inflammatory cytokine secretion, augmenting ICI efficacy (Bellavia et al., 2022; Pouget et al., 2023). 3. Antibody-drug conjugate precision: PSMA-MMAE and similar agents deliver cytotoxic payloads directly to tumor cells while sparing normal tissues, concurrently promoting immune cell infiltration and activation (Lanka et al., 2023).

Early-phase trials demonstrate enhanced ORR and manageable toxicity with 177Lu-PSMA-617 plus PD-1 inhibitors in mCRPC, including a phase I study where pembrolizumab combination therapy achieved superior activity and reduced adverse events compared to monotherapy (Prasad et al., 2021; Aggarwal et al., 2023). These data highlight the potential of PSMA-immune combinatorial strategies to redefine metastatic PCa treatment paradigms.

## 4 Challenges and strategic countermeasures in targeted therapy

While targeted therapies have revolutionized PCa management, inherent challenges—including clonal heterogeneity, adaptive resistance, and tumor evolution—persist, necessitating innovative solutions to optimize therapeutic outcomes.

### 4.1 AR heterogeneity and therapeutic resistance

The AR, a master regulator of male reproductive physiology, exhibits profound heterogeneity across patients and tumor subclones, driven by genetic mutations (e.g., AR-V7 splice variants), post-translational modifications (phosphorylation, acetylation), and epigenetic rewiring (Zamagni et al., 2019; Jaiswal et al., 2022; Kim et al., 2022; Wasim et al., 2022). This variability underpins divergent responses to ADT, with subsets of patients developing resistance through mechanisms such as AR amplification, ligand-independent activation, or glucocorticoid receptor crosstalk (Germain et al., 2020). Paradoxically, AR remains the dominant oncogenic driver in CRPC, yet ARSIs—clinically deployed for over seven decades—yield transient benefits, as most patients progress to CRPC within 12–18 months (Germain et al., 2020). Emerging strategies to circumvent resistance include: 1. Next-generation PROTACs: Advancing beyond first-generation AR degraders, novel dual-target PROTACs are being engineered to simultaneously degrade AR and other key resistance-driving proteins, such as epigenetic regulators (e.g., BRD4) or kinases (e.g., CDK9). This polypharmacological approach can more comprehensively dismantle the oncogenic network and overcome compensatory pathways that lead to single-agent resistance. 2. AR splice variant-specific inhibitors: The AR-V7 variant, which lacks the ligand-binding domain, is a major driver of resistance to conventional antiandrogens. New therapeutic modalities, including small-molecule inhibitors specifically designed to target the unique constitutive activation domain of AR-V7, and monoclonal antibodies that selectively recognize and neutralize AR-V7, are under active investigation to address this critical vulnerability. 3. Subtype-selective AR targeting: Beyond splice variants, development of agents targeting other AR isoforms or specific post-translationally modified AR states. 4. Multimodal combination regimens: Integrating ADT with chemotherapy, radiotherapy, or immune checkpoint inhibitors to exploit synthetic lethality and delay resistance. 5. Epigenetic modulation: Targeting AR co-regulators (e.g., FOXA1, HOXB13) to dismantle compensatory signaling networks. Prospective research must prioritize longitudinal genomic profiling to map AR evolutionary trajectories and identify predictive biomarkers for stratified therapeutic interventions.

### 4.2 Vulnerabilities in alternative splicing and hereditary predisposition

Hereditary predisposition accounts for 10%–20% of PCa cases, with germline mutations in genes such as BRCA2, HOXB13, and MMR pathways contributing to familial clustering (Brandão et al., 2020; Rosellini et al., 2021). Multigene panel testing has identified conserved signaling pathways across hereditary cancers, providing insights into pan-cancer susceptibility mechanisms and enabling molecular stratification to reduce patient heterogeneity (Rosellini et al., 2021). Alternative splicing, a process frequently dysregulated in tumors, disrupts critical pathways involved in drug metabolism, nuclear receptor activation, apoptosis regulation, and immunotherapy response, thereby promoting therapeutic resistance (Ku et al., 2019; Sciarrillo et al., 2020; Li et al., 2023; Seltzer et al., 2023). Clinically, genetic counseling, germline testing, and systematic PSA screening are recommended for high-risk individuals and families to guide early intervention and personalized management (Çelik et al., 2021; Tímár and Uhlyarik, 2022). Addressing splicing-related vulnerabilities and hereditary risk stratification may enhance precision oncology strategies in PCa.

### 4.3 Challenges in selective therapeutic target identification

The development of effective targeted therapies relies on identifying selective molecular targets—proteins or enzymes with which drugs can interact to exert therapeutic effects. However, the complexity and redundancy of biological systems complicate the discovery of such targets, often leading to off-target interactions, unintended systemic effects, and reduced therapeutic efficacy (Dong et al., 2021). Non-selective drug activity not only diminishes clinical outcomes but also poses safety risks, prolongs drug development timelines, and escalates costs. Recent advances have uncovered potential targets through mechanistic studies of prostate carcinogenesis. For instance, circTENM3 suppresses PCa progression by upregulating RUNX3 expression (Janik et al., 2020), while the circSMARCA5/miR-432/PDCD10 axis emerges as a promising therapeutic node via modulation of apoptotic pathways (Lu et al., 2023). Computational approaches, including molecular docking and AI-driven database mining, now accelerate target prediction and drug candidate screening, optimizing preclinical workflows (Ling et al., 2020; Vietri et al., 2021). Additionally, polypharmacological strategies—designing agents that engage multiple targets—may address pathway redundancy while balancing efficacy and toxicity (Chang et al., 2025). These innovations underscore ongoing efforts to overcome target identification barriers and expand precision therapeutic options.

### 4.4 Management of targeted therapy-related adverse effects

The management of adverse effects remains a critical challenge in PCa targeted therapy. While these therapies demonstrate precision in suppressing tumor growth, they often induce systemic toxicities such as gastrointestinal disturbances, immune-related complications, fatigue, hypertension, and hepatotoxicity, which can significantly compromise patient quality of life (Sandhu et al., 2021; Vietri et al., 2021; Zhang et al., 2023). PSMA-targeted radioligand therapies, now established for mCRPC, are under evaluation in earlier disease states, necessitating vigilant monitoring of hematologic and renal parameters (Germain et al., 2020). Similarly, novel ARSIs improve survival in non-castration-resistant metastatic and non-metastatic CRPC but are associated with metabolic and cardiovascular side effects. Optimizing treatment regimens through dose adjustment, preemptive management of predictable toxicities, and enhanced real-time surveillance can mitigate adverse event incidence. Future advancements will rely on prospective clinical trials to refine therapeutic sequencing and combinatorial strategies, aiming to delay resistance while minimizing toxicity. Continued research into molecular mechanisms of drug-related toxicity will further enable the development of safer, more selective agents, ultimately improving the therapeutic index in PCa management.

## 5 Conclusion

Targeted therapies have emerged as a cornerstone of precision oncology in PCa, marked by significant advancements in modulating the AR signaling axis, PI3K/AKT/mTOR pathway, DNA damage repair machinery, and PSMA-directed theranostics. However, the clinical translation of these strategies faces formidable challenges, including AR heterogeneity, spliceosome-driven adaptive resistance, limited target selectivity, and the management of treatment-related adverse events. Addressing these obstacles will require interdisciplinary collaboration, leveraging technologies such as CRISPR-based gene editing, polypharmacological agent design, and artificial intelligence-driven drug discovery to refine therapeutic precision and overcome biological complexity.

Future progress in PCa treatment will depend on integrating mechanistic insights with technological innovation. Future progress will depend on elucidating tumor heterogeneity, optimizing multi-targeted therapeutic regimens, and integrating computational approaches for accelerated drug development. As scientific understanding deepens and translational pipelines mature, the goal of highly personalized, durable PCa management becomes increasingly attainable, potentially enabling metastatic progression to be managed as a chronic condition rather than a terminal diagnosis.

## Abbreviations

AAP, Abiraterone acetate/prednisone; AbEzSvGNPs, Abiraterone-enzalutamide bioconjugated survivin-encapsulated gold nanoparticles; ADT, Androgen Deprivation Therapy; ARSIs, Androgen receptor signaling inhibitors; CRPC, Castration-resistant prostate cancer; DDR, DNA damage repair; DHT, Dihydrotestosterone; HRR, Homologous recombination repair; ICIs, Immune checkpoint inhibitors; MMR, Mismatch repair; MSI, Microsatellite instability; NIRA, Niraparib; NK, Natural killer; ORR, Objective response rates; OS, Overall survival; PCa, Prostate cancer; PROTACs, Proteolysis-targeting chimeras; PSA, Prostate-specific antigen; PSMA, Prostate-specific membrane antigen; RP2D, Recommended phase II dose; TAMs, Tumor-associated macrophages; TILs, tumor-infiltrating lymphocytes; TME, Tumor microenvironment; Tregs, Regulatory T cells.
